# Acute Ischemic Stroke in SARS-CoV, MERS-CoV, SARS-CoV-2: Neurorehabilitation Implications of Inflammation Induced Immunological Responses Affecting Vascular Systems

**DOI:** 10.3389/fneur.2020.565665

**Published:** 2020-12-22

**Authors:** Leila Karimi, Carmela Sales, Sheila Gillard Crewther, Tissa Wijeratne

**Affiliations:** ^1^School of Psychology and Public Health, La Trobe University, Melbourne, VIC, Australia; ^2^Faculty of Social and Political Sciences, Ivane Javakhishvili Tbilisi State University, Tbilisi, Georgia; ^3^Department of Neurology, Australian Institute for Musculoskeletal Science, Level Three, Western Centre for Health Research and Education, Sunshine Hospital, Western Health & University Melbourne, St Albans, VIC, Australia; ^4^Department of Medicine, Faculty of Medicine, University of Rajarata, Anuradhapura, Sri Lanka

**Keywords:** COVID-19, hypercoaguability, ACE—angiotensin-converting enzyme, rehabilitation, SARS, MERS, SARS-CoV2, inflammation

## Abstract

Coronaviruses (CoVs) are enveloped RNA viruses and have been shown to cause mild to severe respiratory infections in humans, with some severe cases inducing neurological manifestations. The lethality and Neurological effects of the Severe Acute Respiratory Syndrome (SARS-CoV), Middle-East Respiratory Syndrome (MERS-CoV), and recently the Severe Acute Respiratory Syndrome coronavirus 2 (SARS-CoV-2) have been well documented though currently there is little literature regarding long term effects and the implications for neurorehabilitation. SARS-CoV-2 and MERS-CoV have been linked to the infection associated inflammatory cytokine storms and induced hypercoagulopathic states that affect the entire vascular system including that of the brain. This mini-review provides an overview of the commonalities among studies published on all three types of the coronavirus related to acute ischemic stroke (AIS). The aim was to elucidate the physiological mechanisms underpinning COVID-2 and to reflect the similarities with the chronic inflammation induced symptoms of AIS that are likely to prove a further challenge for neurorehabilitation clinicians post COVID. In terms of increased incidence of COVID and AIS, it is likely that in depth knowledge of increased thrombotic risk in this population will require appropriate anticoagulation treatment, and other therapeutic interventions as well as neurorehabilitation interventions. Lastly the risk of spreading the virus requires further balancing of the provision of neurorehabiliatation services useful to the patient.

## Introduction

Currently, four genera of *Coronavirina* have been identified, three of which can infect mammals (1). Alpha and beta coronaviruses include the Severe acute respiratory syndrome (SARS-CoV), Middle East respiratory syndrome (MERS-CoV), and Severe acute respiratory syndrome coronavirus 2 (SARS-CoV-2) variants (2, 3). Extensive research in the last two decades has traced the evolutionary origins of these organisms to warm-blooded flying vertebrates where they are non-pathogenic (4). Indeed, a molecular epidemiological study conducted by Woo et al. in 2012 suggests that bats are the most likely sources of coronaviruses and that bats have been instrumental in its widespread dissemination and evolution since 8100 BC (5). The primary entry point of the coronavirus family is the angiotensin-converting enzyme 2 (ACE2) receptor which is ubiquitous across human tissues, especially on epithelial and endothelial cell surfaces. ACE2 plays a vital role in the transmission of the virus as the targeted receptor of the SARS-CoV-2, virus, especially on the epithelia of the respiratory system. Impairment of normal functioning of the ACE2 receptors following the invasion of the virus leads to down regulation of the Renin Angiotensin Aldosterone system (RAAS) and cascade of inflammatory events culminating in pneumonia, severe acute respiratory distress syndrome, multi-organ failure and has led to numerous fatalities during earlier regional outbreaks in China (SARS-CoV), Saudi Arabia (MERS-CoV), and globally SARS-CoV-2 (6). The presence of the ACE2 receptor in other tissues such as the gut, cardiac muscles, and the glial cells of the nervous system contributes to its myriad of manifestations (7–9). Neurological manifestations of coronaviruses were first described during the SARS CoV epidemic in 2002 (10). Tsai et al. reported cases of neuropathies, myopathies, and strokes during the SARS I epidemic while encephalitis and acute inflammatory demyelinating polyneuropathy (AIDP), and Gullian-Barre syndrome (GBS) have also been described in patients with MERS-CoV by Kim et al. (10, 11). Currently, during the SARS-CoV-2 pandemic, an increasing number of patients have presented with stroke and other neurovascular complications as well as the aforementioned manifestations (9, 12).

The following research questions guided this mini review:

- What is the incidence of stroke among patients with SARS, MERS, and SARS-CoV-2?
- What are the main pathophysiological mechanisms of acute ischemic stroke in patients with SARS, MERS, and SARS-CoV-2?
- What are the potential/best treatment options for best clinical outcomes of stroke patients with SARS, MERS, and SARS-CoV-2?
- What is the balance between treating patients while maintaining the case management tailored to avoid the diffusion of infection?

## Methods

### Search Strategy

The concepts of this mini review included studies reporting on the incidence, treatment options, and challenges of ischemic stroke patients with confirmed SARS, MERS, and SARS-CoV-2 infection.

The following search strategy was followed (13):

1. In the first step MEDLINE, Cochrane, and CINAHL databases were searched, followed by title and abstract search.
2. In the second step, the keywords were used when searching on Ovid MEDLINE, Cochrane, PubMed, CINAHL, and EMBASE databases.
3. In the last step, a manual search was carried out to ensure no study was inadvertently left out.

The keywords used to conduct the search were: Stroke, thrombosis, coronavirus, neurorehabilitation, neurological complication, SARS-CoV, MERS-CoV, coronavirus, COVID19, SARS-CoV-2, and autopsy.

### Study Methodologies

Most of the studies included were case reports and case series. Six of the publications were retrospective cohorts while the rest were systematic reviews and commentaries; See Table 1 for key papers.

**Table 1:** Summary of studies on acute ischemic stroke patients with human SARS, MERS, SARS2 (13).

**Author, year, country**	**Title**	**Study design**	**Study population and sample size**	**Key findings**
**SARS-CoV studies**				
Umapathi et al. (Singapore) 2004 (14)	Large artery ischemic stroke in severe acute respiratory syndrome (SARS)	Retrospective cohort	Two hundred and six patients with confirmed SARS	Age ranges of ischemic stroke patients were 39–68. Vascular risk factors were present in 2/5. Five had large artery cerebral infarctions IVIg and anticoagulation was given in 3/5 of the patients 3/5 died
**MERS-CoV studies**				
Arabi et al. (Saudi Arabia) 2015 (15)	Severe neurologic syndrome associated with middle east respiratory syndrome corona virus (MERS-CoV)	Case series	Not applicable	Three patients with confirmed MERS-COV and concomitant neurologic manifestations with one presenting as stroke. 65M DM, HPN with bilateral ICA stenosis. Patient died
Al-Hameed et al. (Saudi Arabia) 2017 (16)	Spontaneous intracranial hemorrhage in a patient with middle east respiratory syndrome corona virus	Case report	Not applicable	42F with confirmed MERS-COV presenting with massive ICH with IV extension. Patient died
Alghatani et al. (Saudi Arabia) 2016 (17)	Neurological complications of middle east respiratory syndrome coronavirus: a report of two cases and review of the literature	Case series	Not applicable	Two patients with confirmed MERS-COV and concomitant neurologic. manifestations with one presenting as stroke. 34F, HPN, DM with intracranial hemorrhage. Patient died
**SARS-CoV2 studies**				
Lodigiania et al. (Italy) 2020 (18)	Venous and arterial thromboembolic complications in COVID-19 patients T admitted to an academic hospital in Milan, Italy	Retrospective cohort	Three hundred and eighty eight consecutive patients with laboratory- proven COVID-19	Eight of the 28 thrombotic complications were acute ischemic stroke. Ages from 64-76. Three of the eight patients had elevated D-dimer. All patients were anticoagulated. 25% of the cases died
Klok et al. (Netherlands) 2020 (19)	Incidence of thrombotic complications in critically ill ICU patients with COVID-19	Retrospective cohort	One hundred and eighty four patients with proven COVID-19 pneumonia	Three of the 31 thrombotic complications were acute ischemic stroke
Mao et al. (China) (9)	Neurologic manifestations of hospitalized patients with coronavirus disease 2019 in Wuhan, China	Retrospective cohort	Two hundred and fourteen patients with coronavirus disease	Six patients had ischemic stroke. Among the severe patients, 4 had ischemia while 1 had ICH. In the non-severe group, 1 had ischemia
Meza et al. (Spain) 2020 (20)	Ischemic stroke in the time of coronavirus disease 2019	Retrospective cohort	Three hundred and fifty four patients with ischemic stroke	Six out of 28 stroke patients tested positive for COVID-19
Aggarwal et al. (USA) 2020 (21)	Cerebrovascular disease is associated with an increased disease severity in patients with coronavirus disease 2019 (COVID-19): a pooled analysis of published literature	Systematic review and metanalysis	Four studies with a sample of 1,829 confirmed COVID-19 patients (553, 30.2% being severe cases)	About 2.6% of 49 patients had a history of cerebrovascular disease or stroke. Experiencing severe COVID-19 was a higher among patients with a history of CV disease (OR = 2.5)
Pranata et al. (Indonesia) 2020 (22)	Impact of cerebrovascular and cardiovascular diseases on mortality and severity of COVID-19—systematic review, meta-analysis, and meta-regression	Systematic review and metanalysis	A total of 4,448 patients were obtained from 16 studies	CV disease was associated with borderline significant for severe COVID-19 [RR 1.88 [1.00, 3.51], *p* = 0.05; *I*^2^: 87%]
Viguier et al. (France) 2020 (23)	Acute ischemic stroke complicating common carotid artery thrombosis during a severe COVID-19 infection	Case report	Not applicable	73F with SARS-COV 2 symptoms 7 days prior to presenting with acute stroke. Thrombus seen at the L CCA
TUNÇ et al. (Turkey) 2020 (24)	Coexistence of Covid-19 and acute ischemic stroke	Case series	Not applicable	Four stroke patients thatdiagnosis of Covid-19 Ages between 45-77. Hypertension was present in 3/4 cases. They presented with COVID symptoms 1–4 days from diagnosis of stroke. Three patients had elevated D-dimer levels, and two of them with high C-reactive protein (CRP).
Oxley et al. (USA) 2020 (25)	Large-vessel stroke as a presenting feature of Covid-19 in the young	Case series	Not applicable	Five stroke patients with diagnosis of SARS-COV2 from USA. Ages are 33–49. Four of the five are males with risk factors of HPN,
				DM and Dyslipidemia. Abnormalities in ferritin, fibrinogen, and D-dimer were noted in most of the patients. Large vessel occlusion were noted in all of the patients with 4/5 undergoing clot retrieval. No deaths were documented
Valderrama et al. (USA) 2020 (26)	Severe acute respiratory syndrome coronavirus 2 infection and ischemic stroke	Case report	Not applicable	52M with HPN with SARS-COV symptoms 7 days prior to presentation. Fibrinogen and D-dimer were elevated. CTA showed ICA occlusion
Beyrouti et al. (UK) 2020 (27)	Characteristics of ischemic stroke associated with COVID-19	Case series	Not applicable	Six stroke patients with diagnosis of SARS-COV2 from UK. Ages are 53–83. Five of the six are males with risk factors of HPN, DM, AF, and Dyslipidemia. Abnormalities in ferritin, fibrinogen and D-dimer were noted in most of the patients. APAS and anticardiolipin antibodies was present in 1 patient while lupus anticoagulant was positive in 5/6. 5/6 patients had large vessel occlusion and most were treated with anticoagulation
Avula et al. (USA) 2020 (12)	COVID-19 presenting as stroke	Case series	Not applicable	Four stroke patients with diagnosis of SARS-COV2 from USA. Ages are 73–88. Three of the four are females with risk factors of HPN, DM, and Dyslipidemia. Leukocytosis was present in 2/4 patients. D-dimer was elevated in 2/4. Large vessel occlusion was seen in 2/4 while stenosis was present in 1/4. Half of the patients died
Berekashvili et al. (USA) 2020 (28)	Etiologic subtypes of ischemic stroke in SARS-COV-2 virus patients	Case series	Not applicable	Ten ischemic stroke patients with diagnosis of SARS-COV2 from USA. Ages are 30–80 years. Six of the 10 are females with risk factors of HPN, DM and Dyslipidemia. Abnormalities in ferritin, fibrinogen and D-dimer were noted in most of the patients. Large vessel occlusion were noted in 5/10 of the patients with three undergoing clot retrieval. Four of the 10 patients died
Moshayedi et al. (USA) 2020 (29)	Triage of acute ischemic stroke in confirmed COVID-19: large vessel occlusion associated with coronavirus infection	Case report	Not applicable	80M with HPN diagnosed with stroke 5 days after experiencing SARS-COV2 symptoms. MRA showed M1 occlusion. Patient was anticoagulated with Heparin but died subsequently
Co et al. (Philippines) 2020 (30)	Intravenous thrombolysis for stroke in a COVID-19 positive filipino patient, a case report	Case report	Not applicable	62F with HPN, DM and dyslipidemia presenting with stroke symptoms 14 days after onset of SARS-COV2 symptoms. Ferritin, D-dimer and CRP were elevated. CTA showed left M1 stenosis. Patient was thrombolysed and discharged with good outcomes
Deliwala et al. (USA) 2020 (31)	Encephalopathy as the sentinel sign of a cortical stroke in a patient infected with coronavirus disease-19 (COVID-19)	Case report	Not applicable	31F with no known vascular risk factors presenting with encephalopathy 5 days prior to experiencing SARS-COV 2 symptoms. CTB showed acute infarct in the right frontal region. She received therapeutic anticoagulation and discharged to rehab subsequently.
Hess et al. (USA) 2020 (32)	COVID-19-related stroke	Commentary	Not applicable	SARS-CoV-1 & 2 deplete ACE2 via receptor endocytosis upon viral entry, leaving ACE1 unopposed with generation of angiotensin II. Angiotensin II worsens lung in- jury and also worsens endothelial function in organs like the heart and brain
Schulman et al. (Canada) 2020 (33)	Coronavirus disease 2019, prothrombotic factors, and venous thromboembolism	Review	Not applicable	Evidence of increased expression in the urokinase pathway involving pro- and antifibrinolytic genes, resulted in increase in plasminogen peptides associated with increased urokinase activity
Amiral et al. (France) 2020 (34)	Covid-19, induced activation of hemostasis, and immune reactions: can an auto-immune reaction contribute to the delayed severe complications observed in some patients?	Review	Not applicable	The delayed autoimmune response contributes to the cytokine storm and generate tissue injury and destruction
Calcagno et al. (Italy) 2020 (35)	Rising evidence for neurological involvement in COVID-19 pandemic	Review	Not applicable	SARS-CoV- binds to the ACE2 receptor on vascular endothelial cells, resulting in abnormally increased blood pressure. Along with platelet and coagulation dysfunctions, the abnormally high blood pressure contributes to the increased risk of intracranial hemorrhage in COVID-19 patients.
Debuc et al. (France) 2020 (36)	Is COVID-19 a new hematologic disease?	Review	Not applicable	The resultant cytokine storm may also cause a surge in interleukin (IL)-2, IL-7, interferon-γ, inducible protein 10, monocyte chemoattractant protein 1, macrophage inflammatory protein 1-α, and tumor necrosis factor-α leading to hyperinflamation. This systemic inflammation causes severe encephalopathy in the patient, and that may lead even to stroke.
Varga et al. (Switzerland) 2020 (37)	Endothelial cell infection and endotheliitis in COVID-19	Case series	Not applicable	Endothelial dysfunction was a main determinant of microvascular dysfunction.
Escalard et al. (France) 2020 (38)	Treatment of acute ischemic stroke due to large vessel occlusion with COVID-19	Case series	Ten patients with confirmed COVID-19 treated for an acute ischemic stroke due to LVO	Best medical care including early intravenous thrombolysis, and successful and prompt recanalization achieved with mechanical thrombectomy, resulted in poor outcomes in patients with COVID-19. 6 patients (60%) died during hospitalization. Despite high angiographic recanalization rates and timeframes, none of our patients had dramatic neurological improvement 24 h after MT.
Zubair et al. (global) 2020 (39)	Neuropathogenesis and neurologic manifestations of the coronaviruses in the age of coronavirus disease 2019: a review	Review	Not applicable	The pathophysiology of increased risk of cerebrovascular disease during COVID-19 infection is likely multifactorial. Common abnormal laboratory test results in patients include elevated leukocyte count, C-reactive protein level, D-dimer level, ferritin level, and lactate dehydrogenase level. 81 Severe cases are characterized by elevated inflammatory markers and hypercoagulability compared with moderate cases and with increased likelihood of stroke.
Benussi et al. (Italy) 2020 (40)	Clinical characteristics and outcomes of inpatients with neurologic disease and COVID-19 in Brescia, Lombardy	Retrospective, single-center cohort study	Fifty six were positive and 117 were negative for COVID-19	Patients with COVID-19 admitted with neurological disease, including stroke, have a significantly higher in-hospital mortality and incident delirium and higher disability than patients without COVID-19

## Results

### The Incidence of Stroke With SARS, MERS, and SARS-COV2

The occurrence of stroke in patients with coronavirus infection was first detailed by Umapathi et al. in four out of the 206 patients with SARS infection in Singapore in 2002. All patients presented with large vessel occlusion (14). Two patients with ischemic stroke (IS) and intracerebral hemorrhage (ICH) were also described in MERS-CoV-infected patients in Saudi Arabia during the 2002 epidemic (15, 17). Most recently, there has been growing concerns about the occurrence of stroke, predominantly affecting large vessels, among confirmed SARS-CoV-2 cases (25). The World Stroke Organization (WSO) has recently identified that stroke increases the severity of SARS-CoV-2 infection by 2.5-fold (21, 41).

While much of the focus has been on cardiovascular, pulmonary, and hematologic complication, there has been a corresponding increase in morbidity and mortality due to neurological complications. According to Bridwell et al. (42), these include acute cerebrovascular events, encephalitis, Guillain-Barré syndrome, acute necrotizing hemorrhagic encephalopathy, and hemophagocytic lymphohistiocytosis (42). Pre-existing neurological conditions have also been linked to more severe COVID-19 infections. It has been documented that elderly patients with chronic medical conditions who contract COVID-19 can present with acute encephalopathy and altered level of consciousness (43).

According to level 1 evidence, “remdesivir therapy in mild to severe disease, and the triple medication regimen (lopinavir-ritonavir, ribavirin, and interferon beta-1b) in mild to moderate disease are promising agents against SARS-CoV-2 in terms of symptom improvement and time to a negative RT-PCR, while further studies are needed (25). However, many of the recently proposed medications, such as antivirals and antimalarials have significant drug interactions and side effects, especially with patients with prior strokes (42). It is important that medical staff are cognizant of potential neurological complications when treating COVID-19. Because some of the neurological manifestations tend to occur early in the illness, neurologists, and neurorehabilitation teams need to be involved, alert, and prepared during the pandemic period (44).

A literature review of neurological manifestations and complications of COVID-19 suggests that most commonly reported symptoms include headache, dizziness, hypogeusia, and neuralgia, followed by stroke, seizures, encephalopathy, and delirium (45, 46). It also highlights that some of the neurological manifestations can precede the typical manifestations such as fever, cough, sore throat, and headaches. The neurological damage caused by COVID-19 can be divided into central and peripheral effects and is likely due to hypoxic brain injury and immune mediated damage to the central nervous system. Currently, the proportion of patients with neurological manifestations is much smaller compared to those with respiratory disease. However, as the pandemic progresses, it is expected that the overall number of patients with neurological symptoms will increase. Neurological complications such as stroke and encephalitis can cause lifelong disability, resulting in long term care needs, associated rehabilitation needs and large health, social and long-term care needs and large health, social, and economic costs (47).

While the incidence of stroke among hospitalized patients with Covid-19 is relatively low, patients in their 30s, 40s, and 50s diagnosed with COVID-19 have presented with large-vessel occlusion stroke (25). Social distancing and isolation are important preventive measures; however, patients with acute neurological symptoms delaying calling an ambulance because of concerns about going to a hospital during the pandemic may contribute to poor outcomes. While strokes occurring in younger, asymptomatic patients with COVID-19 are concerning, patients not seeking care for symptoms of stroke are equally worrying. The overall incidence is low; however, the prognosis of acute ischemic stroke among young adults with COVID-19 is grim. There is evidence that young asymptomatic people are developing clots that cause major stroke. In a small sample, the mortality rate among the COVID-19 patients was 42.8%; the typical mortality from stroke varies between 5 and 10% (45).

Three cases of stroke were also reported during the MERS-CoV epidemic affecting more than 2,500 patients globally so far (48). Majority of the patients were males (66%) older than 45 years (66%). Only one patient was younger than 45 (33%). The average age was 54 years old. The majority had a previous history of HPN (66%), and all suffered from DM (100%). Only one (33%) had a prior history of stroke. All three patients died.

In the most recent SARS-CoV2 pandemic, stroke has been one of the main vascular complications documented. Of the total SARS-CoV2 patients identified in the reported studies, number of males and females are quite comparable; A quarter younger than 45 years, and around half older than 65.

In a university hospital in Italy, among 388 symptomatic and laboratory-confirmed SARS-CoV-2 patients, eight (2%) were diagnosed (after testing positive for COVID) to have ischemic stroke (18). Klok et al. also recorded the occurrence of ischemic stroke in 1.6% (3/188) of all SARS-CoV-2 pneumonia patients admitted in the intensive care unit (ICU) Netherland (19). Neurologic manifestations were also reported in 214 cases in Wuhan, China and among these, five (2.3%) patients presented with ischemic stroke (9). Conversely, Meza et al. reviewed 354 ischemic stroke patients admitted in a local hospital in Italy from December to April and 0.8% of them tested positive for SARS-CoV-2 (20).

While there is increasing evidence of the comorbid occurrence of strokes among patients with coronavirus infection, there is also substantial evidence that pre-existing cerebrovascular disease led to worse clinical outcomes (21, 22). A pooled analysis involving four studies has been performed by Agarwal et al. and has shown that a previous history of stroke increases the severity of SARS-CoV-2 disease by 2.5 times (21). The same trend was observed in another study concluding that cerebrovascular disease among SARS-CoV-2 patients increased the risk of poor outcome and mortality with a relative risk of 2.04 and 2.38, respectively (22).

### The Immunological and Thrombotic Mechanisms

Preliminary evidence shows a pro-coagulatory state associated with COVID-19 infection and development of ischemic stroke (40). There is also evidence that COVID-19 patients admitted with neurological disease, including stroke, have a significantly higher incidence of in-hospital mortality, incident delirium, and higher disability than patients without COVID-19 (49).

Various theories have been proposed as the putative mechanism leading to the increased occurrence of stroke in patients with coronavirus infection. Schulman et al. describe thrombosis to be associated with over activation of the immunosystem and hypercoagulation of the blood through the body including in the brain (33). Another hypothesis proposed is the imbalance in the expression of ACE1/ACE2 as a result of the preferential affinity of the SARS-CoV-2 to ACE2 receptors causing a disproportionate increase in the ACE1/ACE2 balance which has pro-inflammatory and organ damaging effects (32). Thus this mini- review aimed to report on the incidence of stroke among patients with SARS, MERS, and SARS-CoV2- and to elucidate the role of immunological and hypercoagulable mechanisms in acute stroke patients with SARS-CoV, MERS-CoV, and SARS-CoV-2 infection and its pathogenesis.

### Immunological Mechanisms

Various mechanisms have been postulated for the pathogenesis of stroke in patients with coronavirus infection. This includes triggering of the immunological pathways, which results in the activation of the macrophages by the granulocyte–macrophage colony-stimulating factor (GM-CSF) (33). This results in a cytokine surge with a concomitant release of large amounts of Interleukin-6 and other cytokines and chemokines such as interleukin (IL)-2, IL-7, interferon-γ inducible protein 10(IP-10, CXCL10), monocyte chemoattractant protein 1, macrophage inflammatory protein 1-α, and tumor necrosis factor-α (33, 36). These phenomena are reported to be responsible for the cytokine storm triggered by the hyperinflammatory state known to affect blood vessels and further cascade of negative effects in hemostasis leading to vasoconstriction and a prothrombotic state (36, 37). This is further supported by autopsy findings of endotheliitis in patients with SARS-CoV-2 (37). Another hypothesized mechanism is the development of allo-antibodies to ACE2, which also generate a delayed immune response which contributes further to the cytokine storm (34). This state also disrupts the body's physiological capacity to regulate inflammatory and hemostatic processes leading to prothrombotic, pro-inflammatory state and multi-organ damage (34, 50).

### Disruption of the ACE1-ACE2 Balance

Direct attack and damage of the SARS-CoV-2 on the ACE2 receptors of the glial cells of the brain is another putative mechanism for the occurrence of cerebral thrombosis among infected patients (32). Normal activation of ACE-2 is known to be neuroprotective and counteracts the ill effects of the ACE1/angiotensin II/AT1 axis (32). Its depletion favors the activity of the latter, which may lead to a hyperinflammatory state and a cascade of damaging effects to target organs. Direct binding of the virus to the receptors on the endothelial cells may also impair blood pressure control mechanism and oxygen and glucose transfer to cells which may further induce neurological damage (35).

### The Treatment Options and Outcome

Patients comorbid for coronavirus infection and stroke have usually been treated acutely with standard care regimens such as intravenous thrombolysis and endovascular clot retrieval (ECR) as per standard acute stroke guidelines. Three of the five stroke patients during the SARS-CoV pandemic were treated with intravenous immunolglobulin (IVIg). Three out of four MERS-CoV patients were treated with antibiotic and one with IVIg.

Other therapeutic options are currently being investigated including, recombinant ACE2, which may act primarily by competing with he SARS S protein and prevention of the depletion of the receptors (32). Humanized monoclonal antibodies such as tocilizumab and sarilumab, that block the Il-6 receptor have also shown potential in the treatment of vascular complications of human coronavirus (36).

### Neurorehabilitation Challenges

The rapid spread of COVID-19 has become a pandemic emergency departments, choking emergency departments, infectious diseases, and intensive care units. Rehabilitation services have also become affected, causing radical changes both in the organization and in the operating methods (51). Major changes in structure and function of neurorehabilitation and rehabilitation activities during COVID-19 emergency are required, carefully balancing the provision of services useful to the patient and the reduction of the risk of spreading the virus. Stroke in patients with coronavirus infection confers poor clinical outcomes.

## Discussion

Current figures indicate that stroke is not unusual in COVID patients and in fact incidence figures are likely to be a gross underestimation given the novelty of the virus and the fact that imaging is not often undertaken to ascertain cerebro-vascular events. Indeed, confirmatory brain imaging is unusual in COVID-19 patients as they usually require sedation and ventilation.

Eight thousand and ninety-eight cases of SARS infections were reported by WHO during the 2003 outbreak with 10% (774) mortality rate (48). Of these, five patients with an average age of 58 were further identified as suffering a stroke associated with SARS-CoV. Out of these five, three died for a 60% mortality. The high rate of thrombotic complications and the uniform pattern of large vessel ischemic strokes suggested a pro-coagulant state among patients infected with SARS-CoV (14).

While the confirmed cases of MERS-CoV were less than SARS-CoV with around 2,495 cases confirmed worldwide, the reported mortality rate was more than three times higher (585/35%). Three cases of stroke related MERS-CoV were reported. The average age was 54 years and mortality rate of 100% (i.e., 3 out of 3).

To date, COVID-19 has been associated with published cases of stroke among individuals with a mean age of around 65. A quarter of patients were younger than 65 years of age. Hypertension, diabetes, smoking, dyslipidemia are among the reported risk factors for stroke among these patients through in many of these patients a comprehensive stroke work up is not available.

Owing to the immunological nature of the disease, immunoglobulin supplementation has been used in three SARS CoV-1 patients with stroke (14). Currently, there is no published evidence of immunoglobulin use among MERS and SARS-CoV-2 patients. As of July 2020, the majority of COVID-19 patients who also experienced AIS in the US also showed high cerebrovascular risk factors (52). Furthermore, there is still a large gap in the literature regarding underlying mechanisms of stroke in human patients with coronavirus infection.

Preliminary reports (53) suggest that there are three predominant COVID-19- related mechanisms independent of risk factors. These include the hypercoagulable state, vasculitis, and cardiomyopathy (53). It has been postulated that the affinity of the SARS-CoV-2 for ACE2 receptors in the brain allows the virus to damage intracranial arteries, causing vessel wall rupture. Poyiadji et al. (54) suggesting that it is possible that the cytokine storm that accompanies the wall rupture might be the cause of hemorrhagic strokes. This was evident in a COVID-19 patient who developed an acute necrotizing encephalopathy associated with late parenchymal brain hemorrhages. Examining hospitalized patients with COVID-19.

Franceschi et al. (55) found that a combination of cytokine release syndrome and direct SARS-CoV-2–mediated breakdown of the blood–brain barrier may be responsible for hemorrhagic posterior reversible encephalopathy syndrome. In COVID-19 patients, there has also been secondary hemorrhagic transformation of ischemic strokes which may happen in the setting of endothelial damage or a consumption coagulopathy accompanying COVID-19 (53).

Further research is needed to elucidate the role or roles that the activation of the immunological and hypercoagulable pathways and specific risk factors potentially play in underlying susceptibility to coronavirus infection and associated stroke to facilitate the design of better treatment options in the future.

Currently, the total global data on stroke epidemiology during the COVID-19 pandemic is obviously not available. This review provides comprehensive but preliminary assimilation of anecdotal observations, case reports, single center experiences worldwide to date. Anecdotal reports have confirmed a reduction in single event stroke admissions to the hospitals during the global pandemic (47, 56) raising questions whether this is causing more collateral damage by keeping definite mild strokes at home and making these patients more vulnerable to post stroke depression, anxiety, poor quality of life? All these possibilities are hypothetical at present. Only well-designed, prospective research studies will be able to answer these speculations.

### Implications for Recovery and Rehabilitation

As of this writing, there are almost 30 million global cases of COVID-19, with global deaths quickly approaching one million. Over 20 million people have recovered from the disease (57). However, there seems to be very little constructive evaluation of what recovery means in the context of a pandemic where the number of infections continues to increase daily, and a small number of cases of reinfection, possibly via a more recent mutated version of the virus are beginning to appear. Among those reported in the press as no longer hospitalized, there is also a growing number who claim that although home they are far from fully recovered with ongoing problems of fatigue, continuing chest and breathing difficulties and mental health issues (58). What rehabilitation is available to such dehospitalized patients remains to be rigorously investigated.

Lastly, the nature of the virus means that until an effective generalized vaccine exists, the protocols of rehabilitation must change. Social distancing, masks, and telehealth format mean alternate devices for remote continuous physiological monitoring of a patients' health must be made available and incorporated into design of management regimes. In particular, measures of activity, oximetry, heart rate variability, and blood pressure for monitoring of oxygen availability and cardiac output are required during exercise routines and as physiological measures of sleep efficiency that act as surrogate measures of mental health (59).

### Limitations of This Review

This review has been limited to publications in the English language. Most of the publications describe the experience in managing acute stroke patients with SARS-CoV-2, as the far smaller numbers associated with MERS and SARS-CoV necessarily meant there were less comorbid examples of SARS and stroke and hence predominantly case studies. Furthermore, in most cases only patients with ischemic stroke were extensively reviewed, possibly because patients with hemorrhagic strokes died early.

## Conclusion

Patients with COVID-19 exhibit a higher risk of acute ischemic stroke compared with patients with other respiratory tract infections. It is important that medical staff are cognizant of potential neurological complications when treating COVID-19. Health personnel engaged in acute stroke care are at risk of acquiring COVID-19 infection from stroke patients with COVID-19 infection.

There is an increased number of reported strokes during the current COVID-19 pandemic with far greater incidence and apparent contagion rates. Patients with COVID-19 exhibit a higher risk of acute ischemic stroke compared with patients with other respiratory tract infections. In terms of COVID-19 and stroke, greater incidence has been seen in the young where most strokes are associated with large vessel occlusion, and most patients have no previous history of stroke or traditional stroke risk factors. The immunologic and hypercoagulable nature of the disease is displayed by the disproportionate rise in the laboratory markers such as D-dimer, CRP, and ferritin. Standard of care treatment with systemic thrombolysis and endovascular retrieval and therapeutic anticoagulation is being used as the standard treatment at present. However it is beyond the scope of this review to address this aspect in-depth as the COVID-19 pandemic is still unfolding. It is likely that anticoagulation will play an important role in the management of stroke in COVID-19.

As more recovering COVID-19 patients are transferred to rehabilitation services and support, there is a strong need for UpToDate clinician knowledge of increased thrombo-embolic risk in this population. Clinicians need to educate patients about thrombotic events associated with COVID-19 infection. There is also a need to use neuroimaging in the post-acute setting for COVID-19 patients given the prevalence of neurological findings. As bed availability is at a premium and outpatient facilities limited due to the pandemic, access to effective rehabilitation will become challenging.

Another problem for healthcare providers engaged in acute stroke care is the risk of acquiring COVID-19 infection from infected stroke patients. As the best strategy to avoid transmission involves not being in the same space with suspected or confirmed COVID-19 stroke patient, providers should maximize the use of Telestroke or a commercially available low-cost smartphone application system to perform all aspects of acute stroke evaluation.

## Author Contributions

TW, LK, and SC conceived of the presented idea. LK developed the idea further and performed the literature search and drafted the manuscript. CS and TW conducted independent literature search and contributed the PRISMA chart as a group. TW, SC, and CS made significant contributions to the manuscript. All authors approved the final manuscript.

## Conflict of Interest

The authors declare that the research was conducted in the absence of any commercial or financial relationships that could be construed as a potential conflict of interest.
